# A case of eosinophilic cholangitis without bile duct stenosis diagnosed by bile duct biopsy

**DOI:** 10.1002/deo2.108

**Published:** 2022-04-05

**Authors:** Kentaro Ukita, Koichi Izumikawa, Sawako Ishihama, Masashi Nishiyama, Ichiro Sakakihara, Masaki Wato, Koichi Takaguchi

**Affiliations:** ^1^ Postgraduate Clinical Training Center Kagawa Prefectural Central Hospital Kagawa Japan; ^2^ Department of General Medicine Kagawa Prefectural Central Hospital Kagawa Japan; ^3^ Department of Gastroenterology Kagawa Prefectural Central Hospital Kagawa Japan

**Keywords:** bile duct, endoscopic retrograde cholangiopancreatography, eosinophilic cholangitis

## Abstract

Eosinophilic cholangitis (EC) is a rare benign disease that is often misdiagnosed as a malignancy due to the development of biliary stricture. This disease is generally diagnosed by liver biopsy or surgery. Herein, we report a case of EC diagnosed in an 86‐year‐old Japanese woman, who presented with fever, elevated eosinophil count, and elevated liver enzyme level, based on intraductal ultrasound evaluation showing bile duct wall thickening and bile duct biopsy of the same site. We diagnosed this case as EC based on the triad of wall thickening of the biliary system, histopathological findings of eosinophilic infiltration of the biliary tract, and reversibility of biliary abnormalities without treatment. Bile duct biopsy during endoscopic retrograde cholangiopancreatography (ERCP) is rarely used to confirm the diagnosis of EC without bile duct stenosis. For EC and cholecystitis associated with eosinophilia, bile duct biopsy under ERCP, which is less invasive, should be considered. This patient was older than the previously reported patients, and the value of a minimally invasive diagnosis was high.

## INTRODUCTION

Eosinophilic cholangitis (EC) is a rare disease that results in eosinophilic infiltration of the bile duct walls, causing thickening, narrowing, and dilatation of the intrahepatic and extrahepatic bile ducts. There are no diagnostic criteria for this disease, and its pathogenesis is unknown. Although EC is a benign disease, it is often misdiagnosed as cancer due to bile duct stenosis.^1^ Several previous reports confirmed the diagnosis of EC with a liver biopsy; however, there are few reported cases in which diagnosis was confirmed with a bile duct biopsy during endoscopic retrograde cholangiopancreatography (ERCP). Cases wherein the diagnosis was made in the absence of bile duct stenosis are very rare. In this study, we present a case of EC that was pathologically diagnosed by biopsy based on bile duct thickening without stenosis.

## CASE REPORT

An 86‐year‐old Japanese woman presented with fever, fatigue, and breathlessness. Four weeks prior, the patient had a fever of approximately 38°C with an elevated inflammatory response; antibiotics (azithromycin hydrate) were administered. She was referred to our hospital for further investigation, and although her fever had improved, her liver enzyme levels were elevated. Initial blood tests revealed an elevated eosinophil count of 2360/L, aspartate aminotransferase level of 85 U/L, alanine aminotransferase level of 234 U/L, and alkaline phosphatase level of 3870 U/L. We decided to discontinue the antibiotics and observe her conservatively. On the second visit 2 weeks later, the liver enzyme levels were further elevated (Table 1), following which she was admitted to our hospital for further investigation.

**TABLE 1:** Laboratory findings on admission

RBC	2.95 × 10^6^/μl	IgG	1301 mg/dl
Hb	9.2 g/dl	IgG4	75.1 mg/dl
Ht	27.8%	IgE	510 IU/ml
WBC	5.3 × 10^3^/μl	CEA	1.4 ng/ml
Eosinophils	15.5%	CA19‐9	142 U/ml
Plt	34.5 × 10^4^/μl	Anti‐mitochondria M2 antibody	(‐)
TP	6.2 g/dl	MPO‐ANCA	<1.0 U/ml
CRP	1.31 mg/dl	PR3‐ANCA	<1.0 U/ml
T‐Bil	0.4 mg/dl		
D‐Bil	0.2 mg/dl		
AST	285 U/L		
ALT	441 U/L		
ALP	4592 U/L		
γ‐GTP	719 U/L		
LDH	220 U/L		

ALP, alkaline phosphatase; ALT, alanine aminotransferase; AST, aspartate aminotransferase; CA19‐9, carbohydrate antigen 19‐9; CEA, carcinoembryonic antigen; CRP, C‐reactive protein; D‐Bil, direct bilirubin; γ‐GTP, γ‐glutamyl transpeptidase; Hb, hemoglobin; Ht, hematocrit; IgE, immunoglobulin E; IgG, immunoglobulin G; IgG4, immunoglobulin G4; LDH, lactate dehydrogenase; MPO‐ANCA, myeloperoxidase anti‐neutrophil cytoplasmic antibody; Plt, platelet count; PR3‐ANCA, proteinase3 anti‐neutrophil cytoplasmic antibody; RBC, red blood cell; T‐Bil, total bilirubin; TP, total protein; WBC, white blood cell.

**FIGURE 1:**
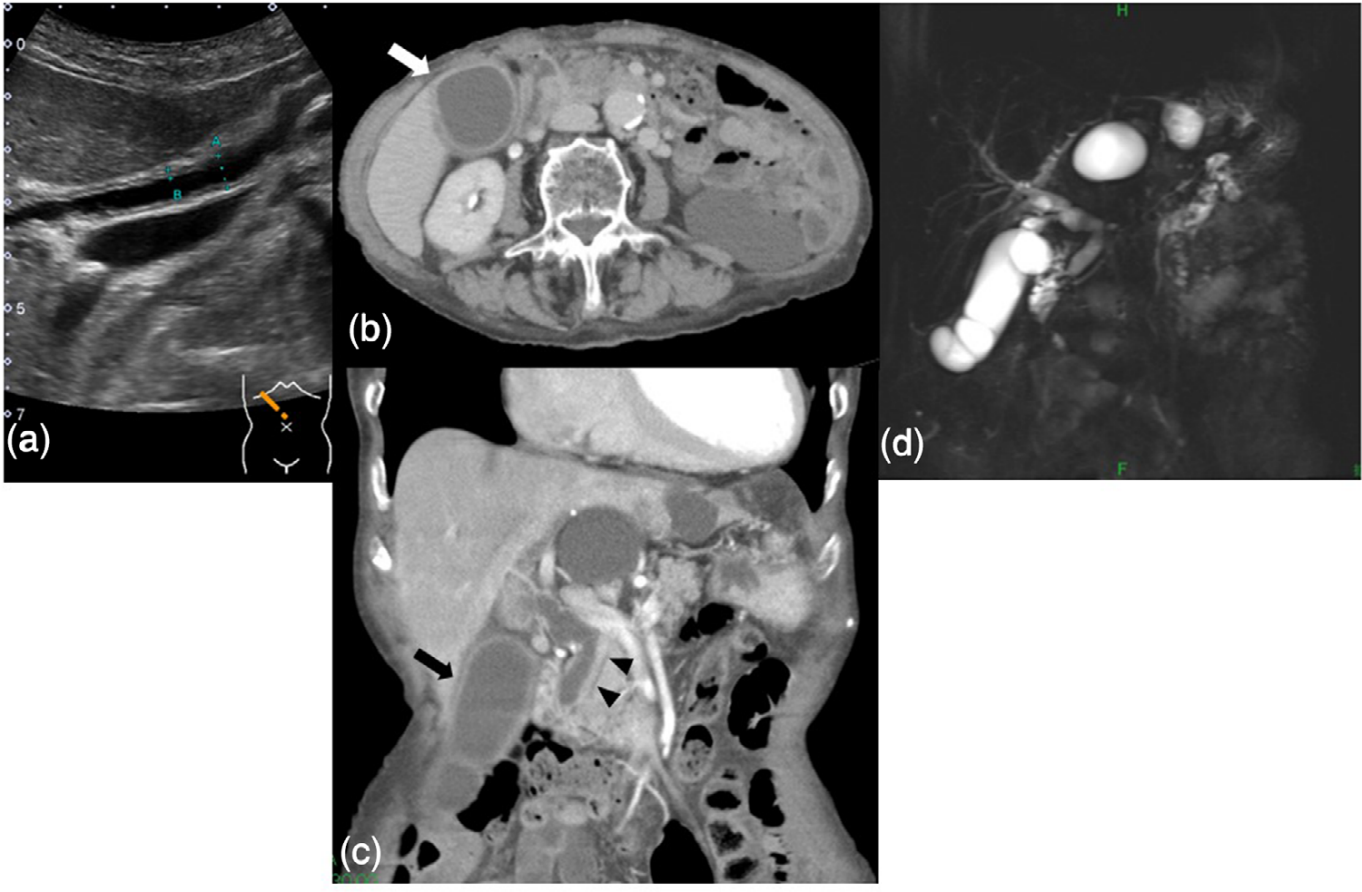
(a) Abdominal ultrasonography showing the diameter of the common bile duct was 6.3 mm (A) and the thickness of the common bile duct wall was 1.3 mm (B). (b, c) Contrast‐enhanced computed tomography of the abdomen showing enlargement and edematous thickening of the gallbladder (→) and bile duct walls (▲). The bile duct dilatation was mild, and no stones were observed. (d) Magnetic resonance cholangiopancreatography showing that the common bile duct was dilated. However, there was no dilatation and stenosis of the intrahepatic bile ducts

**FIGURE 2:**
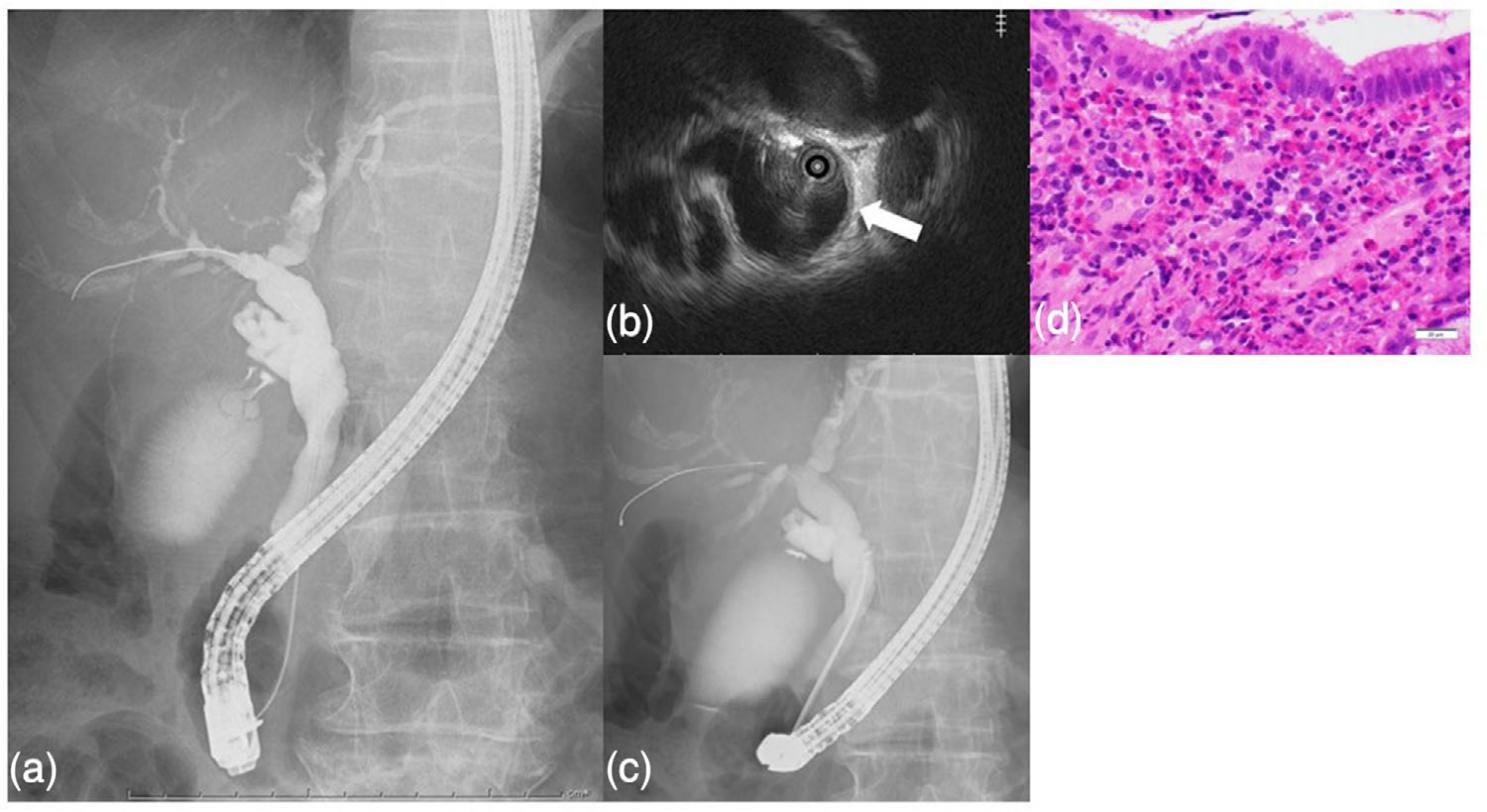
(a) Endoscopic retrograde cholangiopancreatography revealed no sites of stenosis. (b) Intraductal ultrasound indicated diffuse thickening (→) of the extrahepatic and intrahepatic bile ducts. (c) Bile duct biopsy was performed using small cup forceps (Boston Scientific). (d) Histopathological examination showing a collection of eosinophils in the epithelial stroma of the bile duct with no change in the epithelial structure (×400)

Physical examination revealed mild tenderness in the right hypochondriac region. The patient exhibited no symptoms suggestive of neuropathy. She had a history of bronchial asthma but had never traveled abroad or had pets. Abdominal ultrasonography revealed gallbladder and extrahepatic bile duct wall thickening (Figure 1a). Contrast‐enhanced computed tomography (CT) and magnetic resonance cholangiopancreatography (MRCP) showed an enlarged gallbladder with increased wall thickness and dilatation of the common bile duct. There were no stones in the lower bile duct, no dilatation of the intrahepatic bile duct, and no bile duct stenosis (Figure 1b–d). On admission, anti‐neutrophil cytoplasmic antibody, IgG4, and anti‐mitochondrial M2 antibody tests revealed no significant findings. However, the CA19‐9 level (142 U/ml) was abnormally high. The patient needed to be comprehensively evaluated for malignant diseases, primary sclerosing cholangitis, and IgG4‐related diseases. ERCP was performed. Cholangiography revealed no obvious stenosis (Figure 2a).

**FIGURE 3:**
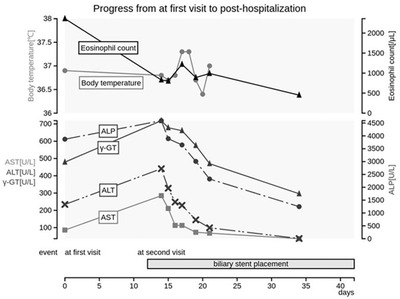
The image shows significant improvement in laboratory values, including eosinophil count, after biliary stent placement, indicating significant progress after the initial examination. ALP, alkaline phosphatase; ALT, alanine aminotransferase; AST, aspartate aminotransferase; γ‐GTP, **γ**‐glutamyl transpeptidase.

The bile color was normal. Intraductal ultrasonography revealed diffuse wall thickening from the extrahepatic bile duct to the intra‐pancreatic bile duct (Figure 2b). Two biopsies were taken from the middle of the bile duct with small cup forceps (Boston Scientific) (Figure 2c), and an endoscopic biliary stent (EBS) 7‐Fr tube was implanted considering the cholestasis. After EBS, both eosinophil count and hepatobiliary enzymes improved (Figure 3) and the patient was discharged from the hospital on day 6 following EBS with no adverse events. The two biliary biopsies revealed eosinophilic infiltration of the bile duct interstitium, with more than 50 and 200 eosinophils per high‐powered microscopic field (HPF) confirming the diagnosis of EC. There were no malignant findings related to the bile duct epithelium (Figure 2d). Better biliary excretion with EBS improved the eosinophil count and hepatobiliary enzyme level in the peripheral blood, and we did not introduce steroids. After the EBS was removed, the patient has been followed up and no further increase in the eosinophil count and no recurrence of wall thickening of the gallbladder or bile ducts on abdominal ultrasonography has been observed since then for 1 year and 4 months.

## DISCUSSION

EC is an uncommon, benign, and self‐limiting cause of biliary stricture characterized by transmural eosinophilic infiltration of the biliary tree that can result in obstructive jaundice.^2^ The pathogenesis of EC has not been fully understood. However, it has been hypothesized that an atopic mechanism may be involved because of the increased number of eosinophils.

In the present case, the cause of EC is unclear. The patient's history of asthma may have had an effect; however, this could not be proven. One case of suspected drug‐induced eosinophilic cholecystitis has been reported.^3^ In the present case, the patient had a fever before the onset of EC and was managed with antibiotics including azithromycin, which is mainly excreted through the hepatobiliary metabolic pathway. Since improved biliary excretion with EBS resulted in the improved eosinophil count and hepatobiliary enzyme level in the peripheral blood, we could not rule out the possibility that the antibiotic played a role in the development of EC in this case.

There are no established diagnostic criteria for EC; however, Matsumoto et al.^4^ proposed the triad of wall thickening or stenosis of the biliary system, histopathological findings of eosinophilic infiltration, and reversibility of biliary abnormalities without treatment or following steroid treatment. The three criteria were met in this case. The thickening of the bile duct wall has been reported to be a characteristic feature of EC on contrast‐enhanced CT and contrast‐enhanced abdominal ultrasonography imaging.^5^ It is impossible to make a diagnosis based solely on these images. According to retrospective studies, two‐thirds of cases of EC are diagnosed after surgery, such as cholecystectomy and cholangiotomy.^6^ Seow‐En et al.^7^ proceeded with surgery for EC with bile duct stenosis, taking into consideration the exclusion of malignancy, recurrence with medical treatment, and side effects of steroid therapy, but it must be noted that EC is a benign disease and surgery is a highly invasive procedure. According to Fukatsu et al.,^8^ there are only seven reported cases of EC that were diagnosed by bile duct biopsy or cytology. Furthermore, the number of infiltrating eosinophils in bile duct biopsies has not been well reported (only three cases), and the number of infiltrating eosinophils ranged from 4 to 60 per HPF.^8^ Two biopsies were taken from the thickened bile duct wall in this case, and the eosinophil counts were greater than 50 and 200/HPF, respectively. There are no established criteria for eosinophil count in the histological diagnosis of EC. More cases of eosinophil infiltration into the bile duct tissues must be collected to establish a quantitative evaluation of biliary eosinophilia. Furthermore, most cases of EC diagnosed by bile duct biopsy are associated with bile duct stricture; except for this case, there is only one case report of EC without biliary stricture.^9^ The common bile duct showed diffuse wall thickening without stenosis in this case and was diagnosed by bile duct biopsy during ERCP, which is a valuable report. When there is a thickening of the bile duct wall and EC is suspected, a bile duct biopsy should be performed, even if there is no bile duct stenosis.

Because of the patient's advanced age, no steroids were introduced, and no specific treatment was given, except for the implantation of the EBS. However, the eosinophil count decreased during the course of the disease and there was no relapse. The bile duct wall was diffusely thickened, suggesting poor drainage of bile due to the edematous thickening of the bile duct wall associated with inflammation. It is considered that EBS resolved the cholestasis, resulting in the improvement of the blood results. In a previous report, the patient was treated solely by drainage,^10^ and the present case supports the validity of this method. But it is unclear whether the improvement in cholestasis alleviated the symptoms or whether EC is a condition that resolves spontaneously. We hope that more cases will be gathered in the future to clarify these questions.

In conclusion, EC is a rare disease and its etiology is unknown. Although the diagnostic criteria have not been established, the presence of eosinophils infiltrating the thickened or narrowed bile duct wall is important for diagnosis. Compared with surgery and liver biopsy, bile duct biopsy under ERCP is less invasive and more useful. If EC is suspected, a biopsy should be performed for diagnostic purposes in thickened bile ducts, even if there is no obvious stricture. However, there are no established criteria for eosinophil count, and more cases need to be accumulated.

## CONFLICT OF INTEREST

The authors declare that they have no conflict of interest.

## FUNDING INFORMATION

None.

## ETHICS STATEMENT

All procedures followed have been performed in accordance with the ethical standards laid down in the 1964 Declaration of Helsinki and its later amendments.

## INFORMED CONSENT

Written informed consent was obtained from the patient for the publication of this case report and the accompanying images.
